# The U‐shape relationship between pulse pressure level on inpatient admission and long‐term mortality in acute coronary syndrome patients undergoing percutaneous coronary intervention

**DOI:** 10.1111/jch.14408

**Published:** 2021-12-09

**Authors:** Huang Wei, Li Hongwei, Sun Ying, Zhang Dai, Wang Man

**Affiliations:** ^1^ Department of Geriatrics and Gerontology Beijing Friendship Hospital Capital Medical University Beijing China; ^2^ Cardiovascular Center Beijing Friendship Hospital Capital Medical University Beijing China

**Keywords:** acute coronary syndrome, all‐cause mortality, cardiac mortality, pulse pressure

## Abstract

The association between pulse pressure and long‐term mortality was investigated among acute coronary syndrome (ACS) patients who received percutaneous coronary intervention (PCI). The study population included 5055 ACS patients in the Department of Cardiology of Beijing Friendship Hospital who were enrolled from January 2013 to July 2019. The median duration of follow‐up was 24 months. Multivariate Cox regression was used to analyze the relationships between PP on inpatient admission and mortalities. Non‐linear associations were studied by restricted cubic splines. Considering the heart function, the analyses were performed in the whole cohort and the LVEF > = 0.5 cohort separately. Subgroup analyses were performed according to the different diagnosis (the myocardial infarction subgroup and the unstable angina pectoris subgroup). When PP was used as categorical variable, the high PP group (≥61 mm Hg) significantly increased the risk of death compared with the intermediate PP group (50–60 mm Hg) in the both cohorts. When PP was used as continuous variable, a U‐shape relationship were found between PP and mortalities in the whole cohort (*p* (for nonlinearity) = .005 and .003, respectively), with reference PP level of 55 mm Hg. However, this U‐shape relationship disappeared in the LVEF > 0.5 cohort (*p* (for nonlinearity) = .111 and .117, respectively). The similar results were obtained in MI subgroup. From this study, the U‐shape relationships between PP level and all‐cause and cardiac mortalities were found in ACS patients who underwent PCI. The U‐shape relationships disappeared in the LVEF > 0.5 cohort. The reference PP level was 55 mm Hg.

## 1 INTRODUCTION

Pulse pressure (PP) is defined as the difference between systolic blood pressure (SBP) and diastolic blood pressure (DBP). PP is the pulsatile component of blood pressure.^1^ Globally, increased PP is related to higher large artery stiffness which leads to an increase in SBP and a decrease in DBP. Increased PP has been reported to be associated with mortality or adverse outcomes in general population,^2^, ^3^, ^4^ hypertensive patients,^5^ myocardial infarction patients,^6^, ^7^ patients with atherothrombosis,^8^ and patients undergoing percutaneous coronary intervention (PCI).^9^ The previous studies also reported the inversely relationship between PP level and adverse outcomes in patients with heart failure (HF),^10^, ^11^ and acute coronary syndrome (ACS).^12^, ^13^ Lower PP may indicate the lower cardiac output. For ACS patients, the U‐shape relationships between PP level and adverse outcomes were found in some studies based on univariate analysis,^14^, ^15^ while the curve often disappeared after multivariable regression analysis. For ACS patients, the prognostic importance of SBP and DBP has been well documented. Nevertheless, the influence of PP on long‐term mortality has been controversially discussed. In the present study, we aimed to examine the association between PP level on inpatient admission and long‐term mortality in ACS patients who underwent PCI.

## 2 METHODS

### 2.1 Study population

**FIGURE 1:**
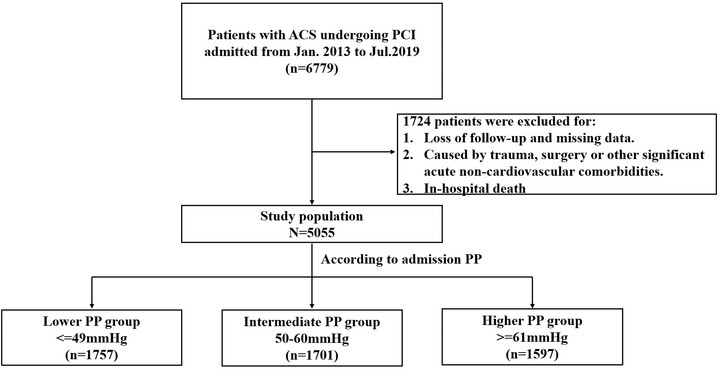
Study flowchart

All consecutive in‐hospital patients with ACS who underwent PCI in the Department of Cardiology of Beijing Friendship Hospital (Beijing, China) from January 2013 to July 2019 were collected. The exclusion criteria were as follows: inaccessible medical records during the follow‐up; ACS was caused by trauma, surgery or other acute non‐cardiovascular comorbidities. The patients who died during hospitalization were excluded. Finally, the study included 5055 patients who received PCI. The patients were divided into tertiles based on PP on inpatient admission: low PP group (≤ 49 mm Hg), intermediate PP group (50‐60 mm Hg), and high PP group (≥ 61 mm Hg). (Figure 1). The study protocol was approved by the Ethics Committee of Beijing Friendship Hospital, Capital Medical University (Approval No. 2020‐P2‐311‐01). The requirement for informed consent was waived for this study.

### 2.2 Data collection and definitions

All data were collected from medical records, including demographic and clinical characteristics (eg, sex, height, weight, heart rate, and blood pressure on admission), medical history, medications, history of interventions, and basic laboratory data. Blood pressure was measured using an automated oscillometric device (OMRON HBP‐1300, OMRON Healthcare Inc., Kyoto, Japan) or a mercury sphygmomanometer (auscultatory method) when patients were immediately admitted in the cardiology department (not in the emergency room or the catheterization laboratory). All patients lay down for at least 5 min in a quiet room before blood pressure measurements. We took three consecutive BP measurements for each arm, and the mean value was obtained. Medical history of the following diseases and history of interventions were obtained: coronary artery disease, myocardial infraction, percutaneous coronary intervention, coronary artery bypass grafting, atrial fibrillation, hypertension, diabetes mellitus, dyslipidemia, and malignancy. Pre–admission and post‐discharge medication data included antiplatelet drugs, statins, β‐blockers, angiotensin‐converting enzyme inhibitors (ACE inhibitors) /angiotensin Ⅱ receptor blockers, calcium channel blockers, and diuretics. Laboratory data included creatinine, albumin, total triglyceride, total cholesterol, hemoglobin A1C, hemoglobin, the peak value of N‐terminal pro‐brain natriuretic peptide (NT‐proBNP) and creatine kinase‐MB.

All patients underwent echocardiography during hospitalization, and the left ventricular ejection fraction (LVEF) was acquired via the modified Simpson method. With reference to the definition of heart failure,^16^ the reduced LVEF was defined as LVEF < 0.5. All patients also underwent coronary angiography and PCI. We categorized patients by the number of diseased vessels (including left anterior descending artery, left circumflex artery, and right coronary artery) with ≥ 50% stenosis in a single, double, triple‐vessel distribution. The left main artery disease was defined as > = 30% stenosis in the left main artery.

PP was defined as SBP minus DBP. The mean arterial pressure (MAP) was defined as [SBP + 2DBP]/3. Body mass index was defined as body mass divided by the square of body height (kg/m^2^). The anemia was defined as hemoglobin < 120 g/L and < 110 g/L in men and women, respectively.^17^ Hypoalbuminemia was defined by a serum albumin level < 35 g/L.^18^

ACS diagnosis criteria were defined according to published guidelines,^19^, ^20^ including UA, NSTEMI, and STEMI. All patients were divided into UA group and MI group (including NSTEMI and STEMI), and the subgroup analyses were performed.

### 2.3 Clinical outcomes

The outcomes examined in this study included all‐cause mortality and cardiac mortality. Cardiac death was defined as death resulting from any cardiac events (eg, myocardial fraction, heart failure, fatal arrhythmia, and sudden death). All‐cause death included both cardiovascular deaths and non‐cardiovascular deaths.

The patients were followed‐up at 1, 3, 6, 12, 24, 36, 48, 60, and 72 months. The outcomes were collected by phone calls or from the inpatient or outpatient medical records during the follow‐ups.

### 2.4 Statistical analysis

Abnormally distributed data of continuous variables were presented as median (interquartile range [IQR]), and were compared by nonparametric tests (eg, the Mann‐Whitney U test). Categorical variables were presented as number/percentage, and were compared using the chi‐squared test or the Fisher's exact test. The univariate Cox analysis was employed for identification of predictors of clinical outcomes (*p *< .05). The covariates include age, sex, ACS diagnosis, atrial fibrillation, diabetes mellitus, malignancy (not for cardiac mortality), current smoker, number of triple‐vessel and left main artery disease, anemia, heart rate, body mass index, creatinine, albumin, and NT‐proBNP peak. Multivariate Cox proportional hazards model (enter method) was used to evaluate the association between PP category and clinical outcome. Hazard ratio (HR) and 95% confidence interval (95% CI) were applied to evaluate the effect. Given the potential non‐linear relationship between PP and mortality, we incorporated restricted cubic splines with three knots as continuous variables to show the shape of the PP‐mortality curve. A *p*‐value of < .05 was considered statistically significant. In order to measure the potential impact of diagnosis on the final results, we performed the subgroup analyses in MI subgroup (including STEMI and NSTEMI) and UA subgroup. All the statistical analyses were performed by SPSS 25.0 software (IBM Corp., Armonk, NY, USA) and R 4.0.2 programming language. Figures were plotted via GraphPad Prism 8.2.1 software (GraphPad Software Inc., San Diego, CA, USA).

## 3 RESULTS

### 3.1 Baseline characteristics

Of the 6779 patients with ACS who underwent PCI in the entire cohort, 1724 patients were excluded. The median duration of follow‐up was 24 (range, 1–82) months. Median age of the patients was 64 (range, 25–93) years old, 71.1% were male, and 51.2% presented with a STEMI or NSTEMI. Baseline characteristics of the study population were listed in **Table** **1**. The correlation of PP level with SBP level was assessed using Pearson's correlation analysis (Pearson's correlation coefficient, 0.80; *p* < .001).

**TABLE 1:** Baseline clinical characteristics of ACS patients on admission according to pulse pressure level

	Low PP group (< = 49 mm Hg) *n* = 1757	Intermediate PP group (50‐60 mm Hg) *n* = 1701	High PP group (> = 61 mm Hg) *n* = 1597	*p*‐value
Age (years)	60.0 (54.0,67.0)	64.0 (58.0,71.0)	67.0 (60.0,76.0)	<.001
Male (*n*, %)	1401 (79.7)	1191 (70.0)	1004 (62.9)	<.001
ACS diagnosis				<.001
STEMI (%)	705 (40.1)	405 (23.8)	331 (20.7)	
NSTEMI (%)	333 (19.0)	348 (20.5)	464 (29.1)	
UA (%)	719 (40.9)	948 (55.7)	802 (50.2)	
History				
Coronary artery disease (%)	787 (44.8)	853 (50.1)	805 (50.4)	.001
Prior myocardial fraction (%)	167 (9.5)	144 (8.5)	135 (8.5)	.459
Prior percutaneous coronary intervention (%)	233 (13.3)	259 (15.2)	176 (17.3)	.005
Prior coronary artery bypass grafting (%)	28 (1.6)	31 (1.8)	38 (2.4)	.238
Atrial fibrillation (%)	74 (4.2)	73 (4.3)	66 (4.1)	.975
Hypertension (%)	981 (55.8)	1172 (68.9)	1287 (80.6)	<.001
Dyslipidemia (%)	873 (49.7)	866 (50.9)	770 (48.2)	.302
Diabetes mellitus (%)	570 (32.4)	656 (38.6)	687 (43.0)	<.001
Malignancy (%)	73 (4.2)	69 (4.1)	69 (4.3)	.930
Current smoker (%)	865 (49.2)	683 (40.2)	540 (33.8)	<.001
Number of narrowed (> = 50%) and obstructed vessels				<.001
1 (single‐vessel, %)	178 (10.1)	128 (7.5)	83 (5.2)	
2 (double‐vessel, %)	295 (16.8)	233 (13.7)	197 (12.3)	
3 (triple‐vessel, %)	1119 (63.7)	1138 (66.9)	1102 (69.0)	
Left main artery disease (%)	165 (9.4)	202 (11.9)	215 (13.5)	
Number of triple‐vessel or left main artery disease (%)	1284 (73.1)	1340 (78.8)	1317 (82.5)	<.001
Pre–admission medical treatment				
Antiplatelet (%)	618 (35.2)	679 (39.9)	623 (39.0)	.010
Beta–blockers (%)	317 (18.0)	354 (2.8)	357 (22.4)	.007
ACE inhibitors/ Angiotensin Ⅱ receptor blockers (%)	455 (25.9)	558 (32.8)	617 (38.6)	<.001
Calcium channel blockers (%)	481 (27.4)	608 (35.7)	712 (44.6)	<.001
Diuretics (%)	81 (4.6)	92 (5.4)	102 (6.4)	.077
Statins (%)	413 (23.5)	491 (28.9)	451 (28.2)	.001
Post–discharge medical treatment				
Antiplatelet (%)	1756 (99.9)	1701 (100.0)	1595 (99.9)	.336
Beta–blockers (%)	1310 (74.6)	1280 (75.2)	1177 (73.7)	.032
ACE inhibitors/ Angiotensin Ⅱ receptor blockers (%)	931 (53.0)	1070 (62.9)	1159 (72.6)	<.001
Calcium channel blockers (%)	282 (16.1)	475 (27.9)	669 (41.9)	<.001
Diuretics (%)	156 (8.9)	132 (7.8)	169 (10.6)	.018
Statins (%)	1646 (93.7)	1615 (94.9)	1494 (93.6)	.168
Heart rate (beats/min)	71.0 (64.0,80.0)	71.0 (64.0,80.0)	70.0 (63.0,78.0)	<.001
SBP (mm Hg)	115.0 (105.0,123.0)	130.0 (121.0,140.0)	147.0 (138.0,158.0)	<.001
DBP (mm Hg)	74.0 (65.0,81.0)	75.0 (70.0,82.0)	74.0 (68.0,81.0)	<.001
MAP (mm Hg)	87.3 (79.0, 95.0)	94.0 (86.7, 100.0)	98.0 (91.3, 106.7)	<.001
Body mass index (kg/m^2^)	25.7 (23.5,27.8)	25.8 (23.8,28.1)	25.6 (23.4,27.9)	.061
Test results				
Creatinine (umol/l) (Ref: Creatinine < 111)	78.80 (68.70,89.20)	77.20 (66.40,90.10)	79.40 (68.20,93.70)	<.001
Creatine kinase‐MB peak (ng/ml) (Ref: Creatine kinase‐MB < 6.6)	5.28 (1.10,110.00)	2.00 (1.00,22.00)	2.60 (1.10,24.10)	<.001
NT‐proBNP peak (ng/l) (Ref: NT‐proBNP < 1800)	561.00 (122.00,2063.00)	311.00 (98.90,1302.00)	453.00 (159.00,1687.00)	<.001
Albumin (g/l) (Ref: Albumin > 35)	38.70 (36.20,41.50)	38.90 (36.60,41.80)	38.40 (35.80,41.00)	<.001
Total triglyceride (mmol/l) (Ref: Total triglyceride < 1.7)	1.41 (1.04,2.04)	1.46 (1.06,2.09)	1.39 (.99,2.01)	.014
Total cholesterol (mmol/l) (Ref: Total cholesterol < 5.2)	4.26 (3.56,4.96)	4.23 (3.58,4.96)	4.23 (3.57,4.92)	.845
Hemoglobin A1C (%) (Ref: Hemoglobin A1C < 6.07)	6.00 (5.50,6.90)	6.20 (5.70,7.20)	6.30 (5.70,7.40)	<.001
Hemoglobin (g/l) (Ref: Hemoglobin < 130)	141.00 (130.00,151.00)	138.00 (127.00,149.00)	134.00 (123.00,146.00)	<.001
LVEF	0.63 (0.55,0.68)	0.65 (0.59,0.69)	0.65 (0.60,0.69)	<.001
LVEF > = 0.5 (%)	1481 (84.3)	1543 (90.7)	1457 (91.2)	<.001
0.5 > LVEF > = 0.4 (%)	206 (11.7)	108 (6.3)	100 (6.3)	
LVEF < 0.4 (%)	70 (4.0)	50 (2.9)	40 (2.5)	
Outcome				
All cause death (%)	72 (4.1)	57 (3.4)	87 (5.4)	.011
Cardiac death (%)	53 (3.0)	36 (2.1)	58 (3.6)	.033

*Abbreviations*: ACS, acute coronary syndrome; DBP, diastolic blood pressure; LVEF, left ventricular ejection fraction; MAP, mean arterial pressure; NT‐proBNP, N‐terminal pro‐brain natriuretic peptide; PCI, prior percutaneous coronary intervention; PP, pulse pressure; SBP, systolic blood pressure; STEMI AND NSTEMI, ST elevation myocardial infarction and non‐ST elevation myocardial infarction; UA, unstable angina pectoris;.

Compared with the low PP group, patients in the high PP group were older, more likely to be female, and had a higher prevalence of coronary artery disease, hypertension, and diabetes mellitus. They also had a higher likelihood of having three arteries or left main coronary artery stenosis after angiography. Because of the higher prevalence of coronary artery disease and hypertension in the high PP group, the patients in the high PP group more frequently took beta blockers, ACE inhibitors/ Angiotensin Ⅱ receptor blockers, and Calcium channel blockers before admission and after discharge. After discharge, there were no differences in taking antiplatelet and statins between groups.

Compared with the other two groups, the patients in the intermediate PP group had a less incidence of STEMI or NSTEMI. They also had lower levels of creatinine, peak Creatine kinase‐MB, peak NT‐proBNP, while higher level of albumin and total triglyceride was detected. In the low PP group, both high hemoglobin level and low LVEF level were detected. During the follow‐ups, the patients in the intermediate PP group experienced lower incidence of all‐cause mortality and cardiac mortality.

### 3.2 Association between PP level and clinical outcomes

**FIGURE 2:**
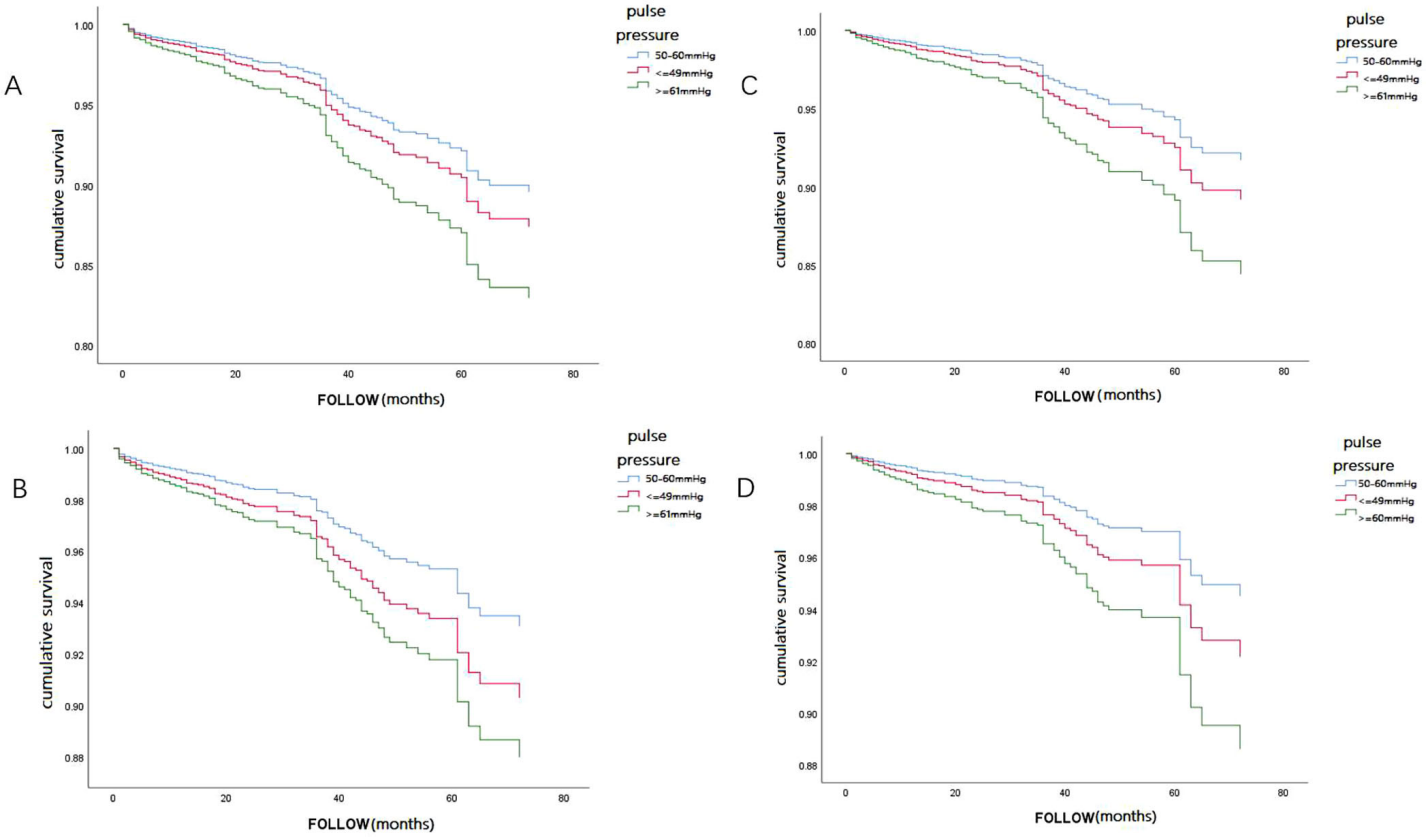
Cumulative survival curves for all‐cause and cardiac mortalities according to PP level on admission. (A): In the whole cohort, for all‐cause mortality. (B): In the whole cohort, for the cardiac mortality. (C): In the LVEF > = 0.5 cohort, for all‐cause mortality. (D): In the LVEF > = 0.5 cohort, for the cardiac mortality

Death occurred in 216 patients, and cardiac death was found in 147 patients. Univariate and multivariate Cox regression analyses were carried out to evaluate the influence of selected covariables on the all‐cause and cardiac mortalities in the whole cohort and LVEF > = 0.5 cohort (Tables S1–S4). Cumulative survival curves for all‐cause and cardiac mortalities in PP‐dependent groups were shown in Figure 2.

Using univariate analysis, PP was significantly associated with the mortalities in both cohorts (*p* < .05). After multivariate analysis, there were statistically significant associations between PP and cardiac mortality in the whole cohort, and between PP and all‐cause mortality in the LVEF > = 0.5 cohort (*p* < .05). In both cohorts, the HRs in the high PP group (≥ 61 mm Hg) for the all‐cause and cardiac mortality were significantly increased compared with the intermediate PP group (50–60 mm Hg). Overall, the ACS patients in the high PP group had an approximately 50% increased hazard of death compared with the intermediate PP group. In the whole cohort, the HR in the low PP group (< = 49 mm Hg) for cardiac mortality was significantly increased compared with the intermediate PP group. However, no statistically significant differences were found when comparing HRs in the low PP group and the intermediate PP group for all‐cause mortality in both cohorts.

Considering the correlation between levels of PP and SBP, multivariate Cox regression analyses were undertaken to investigate the association of SBP level with mortalities. There was no significant association between SBP level and mortality (Tables S5–S8).

**FIGURE 3:**
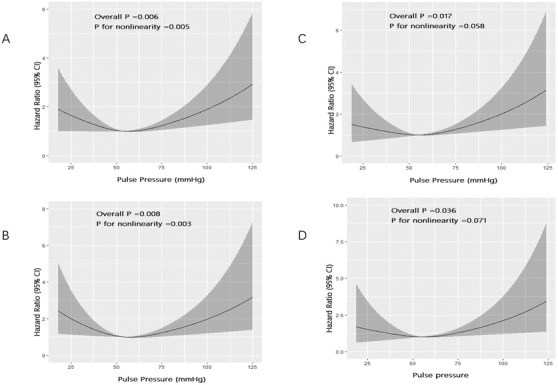
The nonlinear U‐shape relationships between PP level on admission and all‐cause and cardiac mortalities. (A): In the whole cohort, for all‐cause mortality. (B): In the whole cohort, for the cardiac mortality. (C): In the LVEF > = 0.5 cohort, for all‐cause mortality. (D): In the LVEF > = 0.5 cohort, for the cardiac mortality. Data were fitted by a Cox proportional hazards regression model that was based on restricted cubic splines and adjusted for age, sex, ACS diagnosis, atrial fibrillation, diabetes mellitus, malignancy (not for cardiac mortality), smoking history, percentage of three arteries or left main artery involvement, anemia, heart rate, body mass index, creatinine, albumin, and NT‐proBNP peak. Solid black lines represent hazard ratios, and grey shaded areas represent 95% CIs

To further analyze the nonlinear relationships between PP level and mortalities, the restricted cubic spline regression line with three knots was used. The nonlinear U‐shape relationships between PP level on admission and all‐cause and cardiac mortalities were observed in the whole cohort (*p* (for nonlinearity) = .005 and .003, respectively). However, in the LVEF > = 0.5 cohort, the U‐shape relationships were not found (*p* (for nonlinearity) = .058 and .071, respectively). The reference PP level was equal to median PP level (55 mm Hg). The grey shaded area represented the area between the upper level and the lower level of 95% CI. In the LVEF > = 0.5 cohort, the grey shaded area for lower PP level was shifted down compared in the whole cohort. (Figure 3). In the LVEF < 0.5 cohort, there was no significant association between PP level on admission and mortalities. Age and NT‐proBNP level were identified as independent risk factors for mortalities.

### 3.3 Subgroup analysis

There were 1038 myocardial infarction (MI) patients in the low PP group, 753 MI patients in the intermediate PP group, and 795 MI patients in the high PP group. In MI subgroup, PP was the independent risk factor for all‐cause and cardiac mortalities in the both cohorts. The HRs in the high PP group were significantly increased compared with the intermediate PP group (approximately two‐fold). There were no statistically significant differences between the intermediate PP group and the low PP group. The nonlinear U‐shape relationships between PP level and mortalities were observed in the whole cohort (*p* (for nonlinearity) = .009 and .008, respectively). However, in the LVEF > = 0.5 cohort, the U‐shape relationships were not found (*p* (for nonlinearity) = .111 and .117, respectively). Details in Tables S9–S12 and Figure S1.

In the UA subgroup, no significant differences in HR were observed between PP groups. No statistical associations were found among PP and mortalities.

## 4 DISCUSSION

In the present study, we evaluated the correlation of PP level on inpatient admission with long‐term cardiac and all‐cause mortalities in patients with ACS who underwent PCI. The patients with higher PP were likely to be older, female, and had a higher prevalence of hypertension, diabetes mellitus. After angiography, the percentage of patients with triple‐vessel or left main artery disease was highest in the high PP group. The lowest all‐cause and cardiac death rates were found in the intermediate PP group. As a category variable, the HRs for mortalities in the high PP group was significantly increased in both cohorts compared with in the intermediate PP group after multivariate COX regression analyses. As a continuous variable, there were U‐shape relationships between PP level and cardiac and all‐cause mortalities in the whole cohort using 3‐knot restricted cubic spline regression. However, when excluding the effect of deceased heart function, the U‐shape relationships were not observed in the LVEF > = 0.5 cohort. The similar results were found in MI subgroup.

In previous studies, whether lower PP level or higher PP level was the independent predictor of adverse events, the patients with higher PP level were older, and had higher proportion of females, and greater incidence of hypertension and diabetes mellitus, which were consistent with the present study.^21^, ^22^ We also found that patients with higher PP level had a higher proportion of triple‐vessel or left main artery disease. A recent study on PP level and stable angina in patients with multi‐vessel coronary artery disease indicated the similar finding.^23^ Many previous studies have showed higher PP level is associated with a greater risk of total and cardiovascular outcomes in different populations.^2^, ^3^, ^4^, ^6^, ^7^, ^8^, ^21^, ^24^, ^25^, ^26^, ^27^, ^28^ Among the surviving MI patients in the GISSI‐Prevenzione trial, high PP (> 60 mm Hg) were significantly associated with total and cardiovascular mortality after multivariate analysis.^7^ Increased PP might be the result of increased arterial stiffness. The arterial stiffness is influenced by aging, hypertension, and atherosclerosis.^29^ A wider PP may be associated with an increasing cardiac workload and a reduced coronary perfusion,^30^ that can exacerbate myocardial ischemia. In the present study, the patients in the high PP group had an approximately 50% increase in mortalities compared with the intermediate PP group. There were approximately two‐fold increase in MI subgroup.

However, after non‐linear analysis, the U‐shape relationships between PP level and mortalities were observed in the whole cohort, and the U‐shape relationships disappeared in the LVEF > = 0.5 cohort. The area between the upper and lower level of 95% CI for lower PP level was shifted down in the LVEF > = 0.5 cohort compared with in the whole cohort. The change between curves may be explained by the effect of decreased heart function on pulse pressure. PP reflects a complex interaction between intermittent cardiac ejection and dynamic properties of large arteries.^10^ Both cardiac function and arterial stiffness are important components of PP. Numerous studies have demonstrated that a lower PP level is associated with a greater risk of cardiac and/or all‐cause mortality, especially in patients with HF^10^, ^11^, ^22^, ^31^, ^32^ and ACS.^13^ In the Eplerenone Post–Acute Myocardial Infarction Heart Failure Efficacy and Survival Study (EPHESUS) trial, patients with HF and LVEF less than 0.4 were enrolled. It was found that a low PP level was associated with adverse outcome.^10^ PP level was reported to be more dependent on LVEF, rather than being a marker for aortic elasticity.^10^ A low PP level may indicate the low cardiac output, which is the early sign of cardiogenic shock.

As in the previous study,^13^, ^32^ systolic blood pressure demonstrated strong correlation with pulse pressure. In several validated ACS risk scores, SBP‐related variables have been included. In the Thrombolysis in Myocardial Infarction (TIMI) risk score for STEMI, low SBP level (< 100 mm Hg) was a strong risk factor for mortality.^33^ In the Global Registry of Acute Coronary Events (GRACE) risk score, the lower SBP level attained the higher score, indicating the worse outcome.^34^ However, in the present study, the relationships between SBP and mortalities were not found. Similar result about SBP was found in previous study.^32^ Maybe PP is more accurate measure of the cardiac index.^35^ In that study, the adequacy of cardiac output was assessed reliably by PP, not by SBP. There was a poor correlation between cardiac index and SBP.

After subgroup analyses, the significant association between PP and mortalities were only found in MI subgroup. Patients with MI are more likely to suffer from atherosclerotic diseases. And the cardiac function is more susceptible in the MI patients.

Some previous studies also showed the U‐shape relationship between PP level and outcomes in different populations,^14^, ^15^, ^36^, ^37^, ^38^, ^39^ which were mainly formulated by univariate analysis. After adjusted for the confounders, the U‐shape curve disappeared. In the present study, the adjusted U‐shape relationships were found in the whole cohort with the reference PP level of 55 mm Hg. The reference PP level was similar to that in a previous study.^36^

### 4.1 Study limitations

The current study had several limitations. First, this was an observational study conducted in a single‐center, restricting the generalization of the results. Second, we could not obtain data related to blood pressure and medication during follow‐ups. Third, we did not exclude patients who had aortic valve disease, which might influence PP measurement.

## 5 CONCLUSIONS

According to the results of the present study, PP was not only found as an indicator of artery stiffness, but also as an indicator of heart function. In addition to high PP level, low PP level was also associated with increased risk of all‐cause and cardiac mortalities in ACS patients undergoing PCI. Although PP level was correlated with SBP level, SBP level was not found to be linked with the risk of all‐cause and cardiac mortalities. Further studies are warranted to determine the optimal PP level in ACS patients.

## AUTHORS’ CONTRIBUTIONS

L.H. and H.W. designed the study; H.W., Z.D., W.M., S.Y. and L.H. conducted the study; H.W. drafted the manuscript; and L.H. reviewed the manuscript. All the authors read and approved the submitted version of the manuscript

## CONFLICT OF INTEREST

The authors declare that there is no conflict of interest.

## Supporting information

Supplementary information
