# Supplementary material for: The U‐shape relationship between pulse pressure level on inpatient admission and long‐term mortality in acute coronary syndrome patients undergoing percutaneous coronary intervention
Source: J Clin Hypertens (Greenwich). 2021 Dec 9;24(1):58–66. doi: 10.1111/jch.14408 (PMC8783356; doi:10.1111/jch.14408)
Supplement: Supplementary file 1 — Supplementary information [file JCH-24-58-s001.docx]

**Supplemental Table 1** Univariate and multivariate Cox regression analyses of PP level and the all-cause mortality in the whole cohort.

|  | **Univariate** | | | **Multivariate** | | |
| --- | --- | --- | --- | --- | --- | --- |
| **Variable** | **HR** | **95%CI** | **P Value** | **HR** | **95%CI** | **P Value** |
| **Intermediate PP group (50-60mmHg)** | Ref. |  | 0.006 | Ref. |  | 0.058 |
| **Low PP group (<=49mmHg)** | 1.222 | 0.864-1.730 | 0.257 | 1.386 | 0.973-1.976 | 0.071 |
| **High PP group (>=61mmHg)** | 1.695 | 1.214-2.367 | 0.002 | 1.492 | 1.061-2.098 | 0.022 |
| **Age>70 vs Age<=70** | 4.070 | 3.096-5.352 | <0.001 | 2.885 | 2.082-3.999 | <0.001 |
| **Male vs Female** | 0.673 | 0.510-0.888 | 0.005 | 1.029 | 0.758-1.396 | 0.855 |
| **STEMI and NSTEMI vs UA** | 1.448 | 1.092-1.922 | 0.010 | 0.935 | 0.678-1.289 | 0.682 |
| **Atrial fibrillation** | 2.448 | 1.561-3.840 | <0.001 | 1.407 | 0.875-2.264 | 0.159 |
| **Diabetes Mellitus** | 1.310 | 1.000-1.716 | 0.050 | 1.111 | 0.838-1.472 | 0.465 |
| **Malignancy** | 2.128 | 1.258-3.599 | 0.005 | 1.981 | 1.162-3.375 | 0.012 |
| **Current smoker** | 0.675 | 0.509-0.895 | 0.006 | 1.305 | 0.948-1.797 | 0.103 |
| **Number of triple-vessel and left main artery disease** | 2.465 | 1.556-3.905 | <0.001 | 1.814 | 1.137-2.895 | 0.013 |
| **Anemia** | 3.580 | 2.649-4.837 | <0.001 | 1.572 | 1.110-2.224 | 0.011 |
| **Heart rate** | 1.013 | 1.004-1.022 | 0.006 | 1.003 | 0.994-1.013 | 0.495 |
| **Body mass index** | 0.924 | 0.887-0.962 | <0.001 | 0.978 | 0.941-1.016 | 0.258 |
| **Creatinine** | 1.002 | 1.001-1.003 | <0.001 | 1.000 | 0.999-1.002 | 0.669 |
| **Albumin <35g/l vs Albumin >=35g/l** | 2.724 | 2.073-3.581 | <0.001 | 1.265 | 0.918-1.743 | 0.151 |
| **NT-proBNP peak (1000 ng/l increase)** | 1.095 | 1.080-1.110 | <0.001 | 1.066 | 1.044-1.088 | <0.001 |

**Supplemental Table 2** Univariate and multivariate Cox regression analyses of PP level and the cardiac mortality in the whole cohort.

|  | **Univariate** | | | **Multivariate** | | |
| --- | --- | --- | --- | --- | --- | --- |
| **Variable** | **HR** | **95%CI** | **P Value** | **HR** | **95%CI** | **P Value** |
| **Intermediate PP group (50-60mmHg)** | Ref. |  | 0.024 | Ref. |  | 0.044 |
| **Low PP group (<=49mmHg)** | 1.424 | 0.933-2.175 | 0.102 | 1.629 | 1.057-2.511 | 0.027 |
| **High PP group (>=61mmHg)** | 1.786 | 1.178-2.707 | 0.006 | 1.628 | 1.063-2.494 | 0.025 |
| **Age>70 vs Age<=70** | 3.989 | 2.865-5.553 | <0.001 | 2.928 | 1.968-4.356 | <0.001 |
| **Male vs Female** | 0.686 | 0.490-0.961 | 0.029 | 1.025 | 0.705-1.489 | 0.898 |
| **STEMI and NSTEMI vs UA** | 1.834 | 1.283-2.622 | 0.001 | 1.099 | 0.736-1.639 | 0.645 |
| **Atrial fibrillation** | 2.416 | 1.393-4.191 | 0.002 | 1.293 | 0.720-2.322 | 0.389 |
| **Diabetes Mellitus** | 1.478 | 1.068-2.045 | 0.018 | 1.260 | 0.898-1.768 | 0.182 |
| **Current smoker** | 0.723 | 0.515-1.014 | 0.060 | 1.399 | 0.950-2.059 | 0.089 |
| **Number of triple-vessel and left main artery disease** | 3.463 | 1.822-6.582 | <0.001 | 2.509 | 1.309-4.807 | 0.006 |
| **Anemia** | 3.778 | 2.634-5.418 | <0.001 | 1.543 | 1.009-2.357 | 0.045 |
| **Heart rate** | 1.022 | 1.012-1.032 | <0.001 | 1.010 | 0.999-1.021 | 0.066 |
| **Body mass index** | 0.920 | 0.876-0.966 | 0.001 | 0.978 | 0.934-1.024 | 0.344 |
| **Creatinine** | 1.002 | 1.001-1.003 | <0.001 | 1.000 | 0.998-1.002 | 0.998 |
| **Albumin <35g/l vs Albumin >=35g/l** | 2.798 | 2.010-3.896 | <0.001 | 1.163 | 0.791-1.710 | 0.443 |
| **NT-proBNP peak (1000 ng/l increase)** | 1.104 | 1.088-1.122 | <0.001 | 1.071 | 1.047-1.097 | <0.001 |

**Supplemental Table 3** Univariate and multivariate Cox regression analyses of PP level and the all-cause mortality in LVEF>=0.5 cohort.

|  | **Univariate Regression** | | | **Multivariate Regression** | | |
| --- | --- | --- | --- | --- | --- | --- |
| **Variable** | **HR** | **95%CI** | **P Value** | **HR** | **95%CI** | **P Value** |
| **Intermediate PP group (50-60mmHg)** | Ref. |  | 0.004 | Ref. |  | 0.046 |
| **Low PP group (<=49mmHg)** | 1.320 | 0.851-2.046 | 0.215 | 1.563 | 0.998-2.448 | 0.051 |
| **High PP group (>=61mmHg)** | 1.953 | 1.298-2.938 | 0.001 | 1.656 | 1.092-2.511 | 0.018 |
| **Age>70 vs Age<=70** | 3.545 | 2.551-4.927 | <0.001 | 2.740 | 1.848-4.062 | <0.001 |
| **Male vs Female** | 0.689 | 0.492-0.985 | 0.030 | 0.989 | 0.680-1.438 | 0.954 |
| **STEMI and NSTEMI vs UA** | 1.272 | 0.909-1.779 | 0.161 | 0.918 | 0.622-1.354 | 0.666 |
| **Atrial fibrillation** | 2.188 | 1.212-3.951 | 0.009 | 1.340 | 0.724-2.482 | 0.352 |
| **Diabetes Mellitus** | 1.211 | 0.869-1.687 | 0.285 | 1.049 | 0.740-1.486 | 0.789 |
| **Malignancy** | 3.003 | 1.728-5.217 | <0.001 | 2.783 | 1.584-4.890 | <0.001 |
| **Current smoker** | 0.753 | 0.537-1.056 | 0.100 | 1.385 | 0.942-2.036 | 0.098 |
| **Number of triple-vessel and left main artery disease** | 2.270 | 1.330-3.875 | 0.003 | 1.824 | 1.059-3.141 | 0.030 |
| **Anemia** | 3.242 | 2.215-4.744 | <0.001 | 1.655 | 1.081-2.532 | 0.020 |
| **Heart rate** | 1.008 | 0.995-1.020 | 0.237 | 1.007 | 0.994-1.020 | 0.287 |
| **Body mass index** | 0.922 | 0.877-0.969 | 0.001 | 0.967 | 0.922-1.014 | 0.170 |
| **Creatinine** | 1.002 | 1.001-1.003 | 0.001 | 1.001 | 0.999-1.002 | 0.299 |
| **Albumin <35g/l vs Albumin >=35g/l** | 2.478 | 1.763-3.483 | <0.001 | 1.350 | 0.904-2.014 | 0.142 |
| **NT-proBNP peak (1000 ng/l increase)** | 1.087 | 1.065-1.110 | <0.001 | 1.053 | 1.021-1.086 | 0.001 |

**Supplemental Table 4** Univariate and multivariate Cox regression analyses of PP level and the cardiac mortality in LVEF>=0.5 cohort.

|  | **Univariate** | | | **Multivariate** | | |
| --- | --- | --- | --- | --- | --- | --- |
| **Variable** | **HR** | **95%CI** | **P Value** | **HR** | **95%CI** | **P Value** |
| **Intermediate PP group (50-60mmHg)** | Ref. |  | 0.012 | Ref. |  | 0.073 |
| **Low PP group (<=49mmHg)** | 1.443 | 0.832-2.501 | 0.192 | 1.741 | 0.992-3.055 | 0.053 |
| **High PP group (>=61mmHg)** | 2.138 | 1.279-3.574 | 0.004 | 1.770 | 1.048-2.991 | 0.033 |
| **Age>70 vs Age<=70** | 3.813 | 2.533-5.740 | <0.001 | 3.159 | 1.934-5.160 | <0.001 |
| **Male vs Female** | 0.731 | 0.481-1.113 | 0.144 | 1.073 | 0.672-1.714 | 0.768 |
| **STEMI and NSTEMI vs UA** | 1.637 | 1.067-2.513 | 0.024 | 1.153 | 0.708-1.876 | 0.567 |
| **Atrial fibrillation** | 2.563 | 1.289-5.093 | 0.007 | 1.533 | 0.746-3.150 | 0.245 |
| **Diabetes Mellitus** | 1.342 | 0.894-2.014 | 0.156 | 1.227 | 0.802-1.877 | 0.345 |
| **Current smoker** | 0.798 | 0.527-1.208 | 0.286 | 1.455 | 0.905-2.340 | 0.122 |
| **Number of triple-vessel and left main artery disease** | 2.855 | 1.384-5.892 | 0.005 | 2.251 | 1.081-4.685 | 0.030 |
| **Anemia** | 3.349 | 2.170-5.482 | <0.001 | 1.707 | 1.012-2.880 | 0.045 |
| **Heart rate** | 1.015 | 1.001-1.030 | 0.041 | 1.013 | 0.998-1.028 | 0.094 |
| **Body mass index** | 0.916 | 0.861-0.974 | 0.005 | 0.957 | 0.902-1.016 | 0.154 |
| **Creatinine** | 1.002 | 1.001-1.003 | 0.005 | 1.001 | 0.999-1.003 | 0.498 |
| **Albumin <35g/l vs Albumin >=35g/l** | 2.628 | 1.730-3.993 | <0.001 | 1.229 | 0.751-2.011 | 0.413 |
| **NT-proBNP peak (1000 ng/l increase)** | 1.093 | 1.067-1.119 | <0.001 | 1.052 | 1.015-1.091 | 0.006 |

**Supplemental Table 5** Univariate and multivariate Cox regression analyses of SBP level and the all-cause mortality in the whole cohort.

|  | **Univariate** | | | **Multivariate** | | |
| --- | --- | --- | --- | --- | --- | --- |
| **Variable** | **HR** | **95%CI** | **P Value** | **HR** | **95%CI** | **P Value** |
| **Intermediate SBP group (122-139mmHg)** | Ref. |  | 0.418 | Ref. |  | 0.983 |
| **Low SBP group (<=121mmHg)** | 1.019 | 0.728-1.426 | 0.912 | 0.996 | 0.710-1.396 | 0.980 |
| **High SBP group(>=140mmHg)** | 1.217 | 0.875-1.6792 | 0.244 | 1.025 | 0.734-1.431 | 0.886 |
| **Age>70 vs Age<=70** | 4.070 | 3.096-5.352 | <0.001 | 2.894 | 2.094-4.000 | <0.001 |
| **Male vs Female** | 0.673 | 0.510-0.888 | 0.005 | 1.040 | 0.767-1.411 | 0.800 |
| **STEMI and NSTEMI vs UA** | 1.448 | 1.092-1.922 | 0.010 | 0.970 | 0.703-1.338 | 0.853 |
| **Atrial fibrillation** | 2.448 | 1.561-3.840 | <0.001 | 1.418 | 0.882-2.279 | 0.150 |
| **Diabetes Mellitus** | 1.310 | 1.000-1.716 | 0.050 | 1.121 | 0.847-1.485 | 0.425 |
| **Malignancy** | 2.128 | 1.258-3.599 | 0.005 | 1.966 | 1.153-3.352 | 0.013 |
| **Current smoker** | 0.675 | 0.509-0.895 | 0.006 | 1.283 | 0.933-1.763 | 0.125 |
| **Number of triple-vessel and left main artery disease** | 2.465 | 1.556-3.905 | <0.001 | 1.786 | 1.119-2.850 | 0.015 |
| **Anemia** | 3.580 | 2.649-4.837 | <0.001 | 1.586 | 1.122-2.241 | 0.009 |
| **Heart rate** | 1.013 | 1.004-1.022 | 0.006 | 1.002 | 0.993-1.012 | 0.673 |
| **Body mass index** | 0.924 | 0.887-0.962 | <0.001 | 0.976 | 0.939-1.014 | 0.210 |
| **Creatinine** | 1.002 | 1.001-1.003 | <0.001 | 1.000 | 0.999-1.002 | 0.636 |
| **Albumin <35g/l vs Albumin >=35g/l** | 2.724 | 2.073-3.581 | <0.001 | 1.264 | 0.917-1.743 | 0.152 |
| **NT-proBNP peak (1000 ng/l increase)** | 1.095 | 1.080-1.110 | <0.001 | 1.065 | 1.043-1.086 | <0.001 |

**Supplemental Table 6** Univariate and multivariate Cox regression analyses of SBP level and the cardiac mortality in the whole cohort.

|  | **Univariate** | | | **Multivariate** | | |
| --- | --- | --- | --- | --- | --- | --- |
| **Variable** | **HR** | **95%CI** | **P Value** | **HR** | **95%CI** | **P Value** |
| **Intermediate SBP group (122-139mmHg)** | Ref. |  | 0.107 | Ref. |  | 0.255 |
| **Low SBP group (<=121mmHg)** | 1.455 | 0.955-2.217 | 0.081 | 1.429 | 0.936-2.182 | 0.098 |
| **High SBP group(>=140mmHg)** | 1.546 | 1.012-2.362 | 0.044 | 1.258 | 0.819-1.934 | 0.295 |
| **Age>70 vs Age<=70** | 3.989 | 2.865-5.553 | <0.001 | 2.936 | 1.978-4.359 | <0.001 |
| **Male vs Female** | 0.686 | 0.490-0.961 | 0.029 | 1.042 | 0.717-1.512 | 0.831 |
| **STEMI and NSTEMI vs UA** | 1.834 | 1.283-2.622 | 0.001 | 1.148 | 0.769-1.713 | 0.499 |
| **Atrial fibrillation** | 2.416 | 1.393-4.191 | 0.002 | 1.269 | 0.705-2.286 | 0.427 |
| **Diabetes Mellitus** | 1.478 | 1.068-2.045 | 0.018 | 1.268 | 0.905-1.778 | 0.168 |
| **Current smoker** | 0.723 | 0.515-1.014 | 0.060 | 1.379 | 0.938-2.027 | 0.102 |
| **Number of triple-vessel and left main artery disease** | 3.463 | 1.822-6.582 | <0.001 | 2.475 | 1.291-4.746 | 0.006 |
| **Anemia** | 3.778 | 2.634-5.418 | <0.001 | 1.569 | 1.028-2.394 | 0.037 |
| **Heart rate** | 1.022 | 1.012-1.032 | <0.001 | 1.009 | 0.998-1.020 | 0.108 |
| **Body mass index** | 0.920 | 0.876-0.966 | 0.001 | 0.975 | 0.931-1.021 | 0.287 |
| **Creatinine** | 1.002 | 1.001-1.003 | <0.001 | 1.000 | 0.998-1.002 | 0.920 |
| **Albumin <35g/l vs Albumin >=35g/l** | 2.798 | 2.010-3.896 | <0.001 | 1.151 | 0.782-1.694 | 0.476 |
| **NT-proBNP peak (1000 ng/l increase)** | 1.104 | 1.088-1.122 | <0.001 | 1.069 | 1.044-1.094 | <0.001 |

**Supplemental Table 7** Univariate and multivariate Cox regression analyses of SBP level and the all-cause mortality in LVEF>=0.5 cohort.

|  | **Univariate** | | | **Multivariate** | | |
| --- | --- | --- | --- | --- | --- | --- |
| **Variable** | **HR** | **95%CI** | **P Value** | **HR** | **95%CI** | **P Value** |
| **Intermediate SBP group (122-139mmHg)** | Ref. |  | 0.087 | Ref. |  | 0.347 |
| **Low SBP group (<=121mmHg)** | 0.703 | 0.409-1.205 | 0.200 | 1.034 | 0.664-1.610 | 0.881 |
| **High SBP group(>=140mmHg)** | 1.262 | 1.796-2.001 | 0.323 | 1.299 | 1.891-1.894 | 0.173 |
| **Age>70 vs Age<=70** | 3.545 | 2.551-4.927 | <0.001 | 2.672 | 1.810-3.944 | <0.001 |
| **Male vs Female** | 0.689 | 0.492-0.985 | 0.030 | 0.993 | 0.682-1.445 | 0.971 |
| **STEMI and NSTEMI vs UA** | 1.272 | 0.909-1.779 | 0.161 | 0.975 | 0.660-1.441 | 0.900 |
| **Atrial fibrillation** | 2.188 | 1.212-3.951 | 0.009 | 1.392 | 0.755-2.569 | 0.290 |
| **Diabetes Mellitus** | 1.211 | 0.869-1.687 | 0.285 | 1.041 | 0.736-1.472 | 0.820 |
| **Malignancy** | 3.003 | 1.728-5.217 | <0.001 | 2.810 | 1.599-4.939 | <0.001 |
| **Current smoker** | 0.753 | 0.537-1.056 | 0.100 | 1.369 | 0.932-2.010 | 0.109 |
| **Number of** **triple-vessel and left main artery disease** | 2.270 | 1.330-3.875 | 0.003 | 1.756 | 1.019-3.025 | 0.042 |
| **Anemia** | 3.242 | 2.215-4.744 | <0.001 | 1.703 | 1.117-2.598 | 0.013 |
| **Heart rate** | 1.008 | 0.995-1.020 | 0.237 | 1.005 | 0.992-1.017 | 0.442 |
| **Body mass index** | 0.922 | 0.877-0.969 | 0.001 | 0.963 | 0.918-1.011 | 0.128 |
| **Creatinine** | 1.002 | 1.001-1.003 | 0.001 | 1.001 | 0.999-1.002 | 0.291 |
| **Albumin <35g/l vs Albumin >=35g/l** | 2.478 | 1.763-3.483 | <0.001 | 1.337 | 0.897-1.992 | 0.154 |
| **NT-proBNP peak (1000 ng/l increase)** | 1.087 | 1.065-1.110 | <0.001 | 1.052 | 1.020-1.085 | 0.001 |

**Supplemental Table 8** Univariate and multivariate Cox regression analyses of SBP level and the cardiac mortality in LVEF>=0.5 cohort.

|  | **Univariate** | | | **Multivariate** | | |
| --- | --- | --- | --- | --- | --- | --- |
| **Variable** | **HR** | **95%CI** | **P Value** | **HR** | **95%CI** | **P Value** |
| **Intermediate SBP group (122-139mmHg)** | Ref. |  | 0.012 | Ref. |  | 0.180 |
| **Low SBP group (<=121mmHg)** | 1.443 | 0.832-2.501 | 0.192 | 1.525 | 0.891-2.611 | 0.124 |
| **High SBP group(>=140mmHg)** | 2.138 | 1.279-3.574 | 0.004 | 1.519 | 0.933-2.473 | 0.093 |
| **Age>70 vs Age<=70** | 3.813 | 2.533-5.740 | <0.001 | 3.134 | 1.927-5.099 | <0.001 |
| **Male vs Female** | 0.731 | 0.481-1.113 | 0.144 | 1.061 | 0.663-1.698 | 0.805 |
| **STEMI and NSTEMI vs UA** | 1.637 | 1.067-2.513 | 0.024 | 1.194 | 0.733-1.946 | 0.476 |
| **Atrial fibrillation** | 2.563 | 1.289-5.093 | 0.007 | 1.563 | 0.761-3.210 | 0.224 |
| **Diabetes Mellitus** | 1.342 | 0.894-2.014 | 0.156 | 1.224 | 0.802-1.868 | 0.348 |
| **Current smoker** | 0.798 | 0.527-1.208 | 0.286 | 1.467 | 0.911-2.362 | 0.115 |
| **Number of triple-vessel and left main artery disease** | 2.855 | 1.384-5.892 | 0.005 | 2.199 | 1.056-4.582 | 0.035 |
| **Anemia** | 3.349 | 2.170-5.482 | <0.001 | 1.785 | 1.064-2.993 | 0.028 |
| **Heart rate** | 1.015 | 1.001-1.030 | 0.041 | 1.011 | 0.996-1.025 | 0.149 |
| **Body mass index** | 0.916 | 0.861-0.974 | 0.005 | 0.955 | 0.900-1.014 | 0.136 |
| **Creatinine** | 1.002 | 1.001-1.003 | 0.005 | 1.001 | 0.999-1.003 | 0.454 |
| **Albumin <35g/l vs Albumin >=35g/l** | 2.628 | 1.730-3.993 | <0.001 | 1.205 | 0.739-1.965 | 0.455 |
| **NT-proBNP peak (1000 ng/l increase)** | 1.093 | 1.067-1.119 | <0.001 | 1.049 | 1.012-1.087 | 0.010 |

**Supplemental Table 9** Univariate and multivariate Cox regression analyses of PP level and the all-cause mortality in the whole cohort in MI subgroup

|  | **Univariate** | | | **Multivariate** | | |
| --- | --- | --- | --- | --- | --- | --- |
| **Variable** | **HR** | **95%CI** | **P Value** | **HR** | **95%CI** | **P Value** |
| **Intermediate PP group (50-60mmHg)** | Ref. |  | 0.002 | Ref. |  | 0.015 |
| **Low PP group (<=49mmHg)** | 1.179 | 0.749-1.858 | 0.477 | 1.481 | 0.932-2.352 | 0.096 |
| **High PP group (>=61mmHg)** | 2.002 | 1.296-3.092 | 0.002 | 1.933 | 1.239-3.018 | 0.004 |
| **Age>70 vs Age<=70** | 4.681 | 3.326-6.588 | <0.001 | 3.427 | 2.256-5.204 | <0.001 |
| **Male vs Female** | 0.650 | 0.456-0.926 | 0.017 | 1.199 | 0.811-1.774 | 0.363 |
| **Atrial fibrillation** | 2.207 | 1.192-4.048 | 0.012 | 1.105 | 0.572-2.136 | 0.766 |
| **Diabetes Mellitus** | 1.541 | 1.101-2.155 | 0.012 | 1.208 | 0.851-1.714 | 0.290 |
| **Malignancy** | 1.822 | 0.851-3.898 | 0.122 | 1.754 | 0.813-3.787 | 0.152 |
| **Current smoker** | 0.644 | 0.460-0.902 | 0.010 | 1.447 | 0.986-2.124 | 0.059 |
| **Number of triple-vessel and left main artery disease** | 2.438 | 1.404-4.233 | 0.002 | 1.670 | 0.951-2.930 | 0.074 |
| **Anemia** | 3.319 | 2.298-4.813 | <0.001 | 1.323 | 0.856-2.043 | 0.208 |
| **Heart rate** | 1.011 | 1.001-1.022 | 0.040 | 1.004 | 0.993-1.016 | 0.462 |
| **Body mass index** | 0.933 | 0.889-0.980 | 0.006 | 0.992 | 0.947-1.040 | 0.747 |
| **Creatinine** | 1.002 | 1.001-1.003 | <0.001 | 1.000 | 0.998-1.002 | 0.976 |
| **Albumin <35g/l vs Albumin >=35g/l** | 2.399 | 1.725-3.337 | <0.001 | 1.148 | 0.794-1.659 | 0.462 |
| **NT-proBNP peak (1000 ng/l increase)** | 1.088 | 1.071-1.106 | <0.001 | 1.068 | 1.044-1.093 | <0.001 |

**Supplemental Table 10** Univariate and multivariate Cox regression analyses of PP level and the cardiac mortality in the whole cohort in MI subgroup.

|  | **Univariate** | | | **Multivariate** | | |
| --- | --- | --- | --- | --- | --- | --- |
| **Variable** | **HR** | **95%CI** | **P Value** | **HR** | **95%CI** | **P Value** |
| **Intermediate PP group (50-60mmHg)** | Ref. |  | 0.007 | Ref. |  | 0.015 |
| **Low PP group (<=49mmHg)** | 1.374 | 0.799-2.360 | 0.251 | 1.734 | 0.998-3.012 | 0.051 |
| **High PP group (>=61mmHg)** | 2.190 | 1.295-3.701 | 0.002 | 2.215 | 1.293-3.796 | 0.004 |
| **Age>70 vs Age<=70** | 4.319 | 2.908-6.413 | <0.001 | 3.501 | 2.155-5.687 | <0.001 |
| **Male vs Female** | 0.744 | 0.487-1.138 | 0.173 | 1.355 | 0.845-2.173 | 0.207 |
| **Atrial fibrillation** | 2.192 | 1.065-4.510 | 0.033 | 0.995 | 0.457-2.168 | 0.991 |
| **Diabetes Mellitus** | 1.812 | 1.230-2.668 | 0.003 | 1.400 | 0.933-2.099 | 0.104 |
| **Current smoker** | 0.699 | 0.473-1.032 | 0.072 | 1.567 | 1.001-2.452 | 0.049 |
| **Number of triple-vessel and left main artery disease** | 3.705 | 1.720-7.978 | 0.001 | 2.507 | 1.151-5.459 | 0.021 |
| **Anemia** | 3.352 | 2.173-5.169 | <0.001 | 1.259 | 0.750-2.112 | 0.383 |
| **Heart rate** | 1.020 | 1.009-1.031 | <0.001 | 1.013 | 1.000-1.025 | 0.044 |
| **Body mass index** | 0.938 | 0.886-0.994 | 0.029 | 0.999 | 0.946-1.056 | 0.982 |
| **Creatinine** | 1.002 | 1.001-1.003 | <0.001 | 1.000 | 0.998-1.002 | 0.871 |
| **Albumin <35g/l vs Albumin >=35g/l** | 2.196 | 1.492-3.232 | <0.001 | 1.053 | 0.683-1.623 | 0.816 |
| **NT-proBNP peak (1000 ng/l increase)** | 1.093 | 1.073-1.113 | <0.001 | 1.070 | 1.043-1.099 | <0.001 |

**Supplemental Table 11** Univariate and multivariate Cox regression analyses of PP level and the all-cause mortality in LVEF>=0.5 cohort in MI subgroup.

|  | **Univariate Regression** | | | **Multivariate Regression** | | |
| --- | --- | --- | --- | --- | --- | --- |
| **Variable** | **HR** | **95%CI** | **P Value** | **HR** | **95%CI** | **P Value** |
| **Intermediate PP group (50-60mmHg)** | Ref. |  | 0.005 | Ref. |  | 0.025 |
| **Low PP group (<=49mmHg)** | 1.303 | 0.713-2.380 | 0.389 | 1.734 | 0.932-3.228 | 0.082 |
| **High PP group (>=61mmHg)** | 2.310 | 1.318-4.051 | 0.003 | 2.220 | 1.248-3.951 | 0.007 |
| **Age>70 vs Age<=70** | 4.182 | 2.734-6.398 | <0.001 | 3.683 | 2.178-6.228 | <0.001 |
| **Male vs Female** | 0.665 | 0.424-1.041 | 0.074 | 1.101 | 0.663-1.827 | 0.710 |
| **Atrial fibrillation** | 1.781 | 0.722-4.393 | 0.210 | 0.854 | 0.325-2.247 | 0.749 |
| **Diabetes Mellitus** | 1.328 | 0.860-2.050 | 0.201 | 1.159 | 0.737-1.822 | 0.522 |
| **Malignancy** | 2.547 | 1.110-5.843 | 0.027 | 2.636 | 1.132-6.138 | 0.025 |
| **Current smoker** | 0.780 | 0.512-1.187 | 0.246 | 1.656 | 1.027-2.671 | 0.038 |
| **Number of triple-vessel and left main artery disease** | 1.994 | 1.060-3.751 | 0.032 | 1.531 | 0.802-2.922 | 0.197 |
| **Anemia** | 2.669 | 1.606-4.435 | <0.001 | 1.271 | 0.722-2.240 | 0.406 |
| **Heart rate** | 1.009 | 0.994-1.024 | 0.227 | 1.011 | 0.996-1.027 | 0.152 |
| **Body mass index** | 0.945 | 0.888-1.006 | 0.076 | 0.992 | 0.933-1.054 | 0.788 |
| **Creatinine** | 1.002 | 1.000-1.003 | 0.013 | 1.001 | 0.999-1.003 | 0.539 |
| **Albumin <35g/l vs Albumin >=35g/l** | 2.173 | 1.426-3.312 | <0.001 | 1.207 | 0.751-1.941 | 0.437 |
| **NT-proBNP peak (1000 ng/l increase)** | 1.078 | 1.052-1.105 | <0.001 | 1.054 | 1.018-1.092 | 0.003 |

**Supplemental Table 12** Univariate and multivariate Cox regression analyses of PP level and the cardiac mortality in LVEF>=0.5 cohort in MI subgroup.

|  | **Univariate** | | | **Multivariate** | | |
| --- | --- | --- | --- | --- | --- | --- |
| **Variable** | **HR** | **95%CI** | **P Value** | **HR** | **95%CI** | **P Value** |
| **Intermediate PP group (50-60mmHg)** | Ref. |  | 0.012 | Ref. |  | 0.043 |
| **Low PP group (<=49mmHg)** | 1.443 | 0.832-2.501 | 0.192 | 2.059 | 0.972-4.362 | 0.059 |
| **High PP group (>=61mmHg)** | 2.138 | 1.279-3.574 | 0.004 | 2.450 | 1.212-4.956 | 0.013 |
| **Age>70 vs Age<=70** | 3.813 | 2.533-5.740 | <0.001 | 3.922 | 2.119-7.258 | <0.001 |
| **Male vs Female** | 0.731 | 0.481-1.113 | 0.144 | 1.101 | 0.402-3.016 | 0.200 |
| **Atrial fibrillation** | 2.563 | 1.289-5.093 | 0.007 | 1.470 | 0.871-2.481 | 0.851 |
| **Diabetes Mellitus** | 1.342 | 0.894-2.014 | 0.156 | 1.227 | 0.802-1.877 | 0149 |
| **Current smoker** | 0.798 | 0.527-1.208 | 0.286 | 1.858 | 1.058-3.262 | 0.031 |
| **Number of triple-vessel and left main artery disease** | 2.855 | 1.384-5.892 | 0.005 | 2137 | 0.906-5.038 | 0.083 |
| **Anemia** | 3.349 | 2.170-5.482 | <0.001 | 1.310 | 0.669-2.565 | 0.431 |
| **Heart rate** | 1.015 | 1.001-1.030 | 0.041 | 1.020 | 1.003-1.037 | 0.019 |
| **Body mass index** | 0.916 | 0.861-0.974 | 0.005 | 0.997 | 0.927-1.073 | 0.940 |
| **Creatinine** | 1.002 | 1.001-1.003 | 0.005 | 1.001 | 0.999-1.003 | 0.419 |
| **Albumin <35g/l vs Albumin >=35g/l** | 2.628 | 1.730-3.993 | <0.001 | 1.203 | 0.684-2.118 | 0.521 |
| **NT-proBNP peak (1000 ng/l increase)** | 1.093 | 1.067-1.119 | <0.001 | 1.051 | 1.008-1.095 | 0.020 |


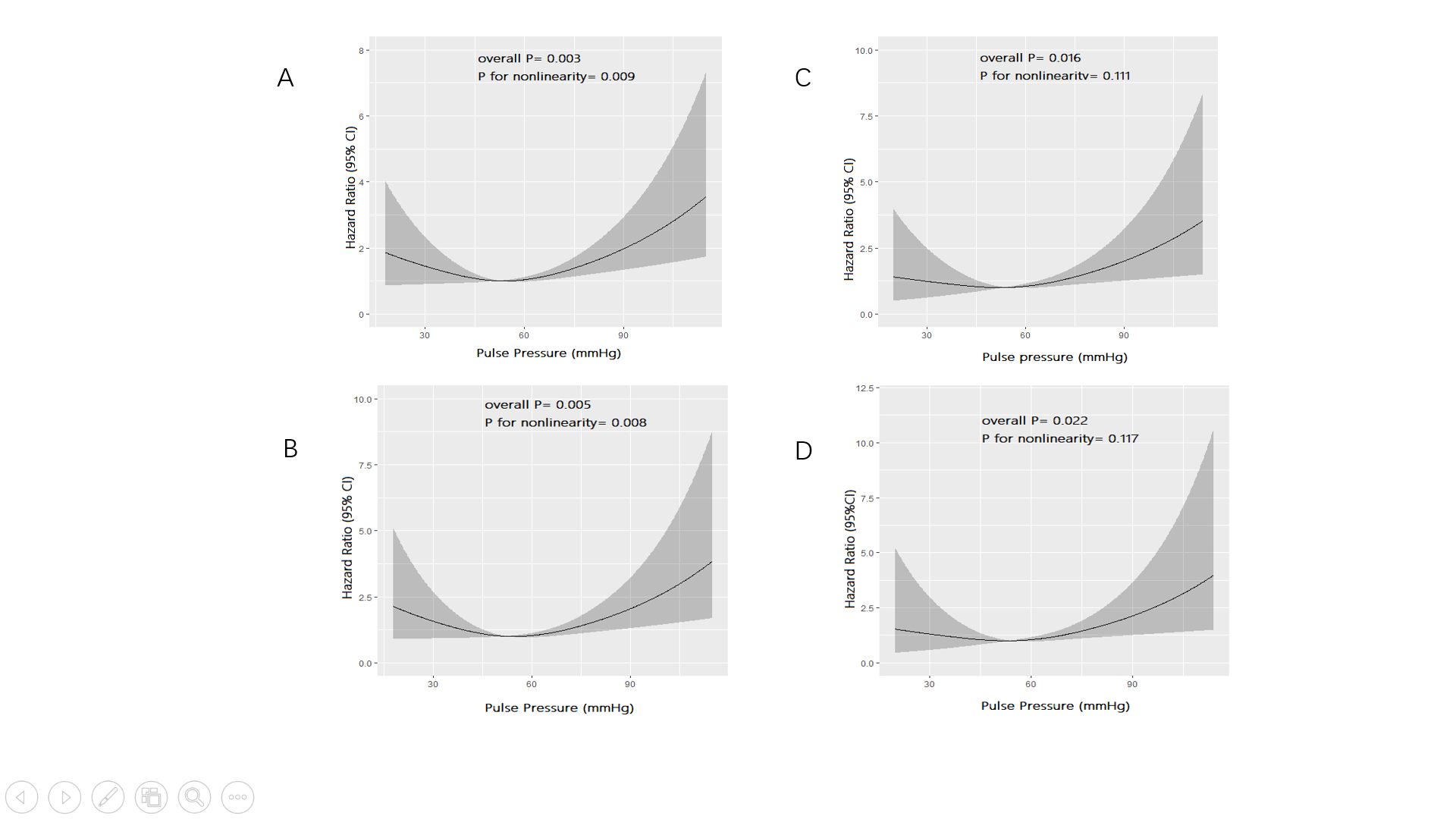


**Supplemental Figure 1** The nonlinear U-shape relationships between PP level on admission and all-cause and cardiac mortalities in MI subgroup analysis.

A: In the whole cohort, for all-cause mortality. B: In the whole cohort, for the cardiac mortality.

C: In the LVEF>=0.5 group, for all-cause mortality. D: In the LVEF>=0.5 group, for the cardiac mortality.

a Data were fitted by a Cox proportional hazards regression model that was based on restricted cubic splines and adjusted for age, gender, ACS diagnosis, atrial fibrillation, diabetes mellitus, malignancy (not for cardiac mortality), smoking history, percentage of three arteries or left main artery involvement, anemia, heart rate, body mass index, creatinine, albumin, and NT-proBNP peak.

Solid black lines represent hazard ratios, and grey shaded areas represent 95% CIs.
